# Elemental and Morphological Mapping of Enamel Surface to Predict the Potential Impact of Charcoal-Based Dentifrices

**DOI:** 10.1021/acsomega.5c11029

**Published:** 2026-03-23

**Authors:** Marian P. Alvarenga, Luís Eduardo S Soares

**Affiliations:** Laboratory of Dentistry and Applied Materials, Research and Development Institute, 67655 University of Vale do Paraíba, Sao Jose dos Campos 12244-000, Brazil

## Abstract

This in vitro study aimed to assess the effects of the inorganic elements in charcoal-based dentifrices on the bovine enamel in terms of surface morphology and composition. Enamel surfaces (*n* = 10/group) obtained from bovine incisors were brushed with the conventional toothpaste Oral-B Complete (OBC; negative control), the whitening toothpaste Colgate Luminous White Expert (LWE; positive control), and the activated charcoal-based dentifrices Colgate Natural Extracts Purifying (CNE), Curaprox Black Is White (CBW), and Oral-B 3D White Mineral Clean (OBW). Enamel samples were analyzed using microenergy-dispersive X-ray fluorescence spectrometry (μ-EDXRF), surface roughness (*R*_a_), contact angle (CA), and scanning electron microscopy (SEM). FT-Raman and Fourier-Transform Infrared (FTIR) spectroscopy and SEM were used to analyze the particulate fractions of the dentifrices. Enamel *R*_a_ varied between 0.029 (0.006) and 0.037 (0.006) and demonstrated no significant between-group difference. The CA values ranged from 90.59 (13.16) in the OBC to 102.6 (13.32) in the CBW, with no significance. μ-EDXRF revealed significant mineral loss with the OBC and CNE, while SEM confirmed deeper abrasion patterns linked to larger heterogeneous particles. FT-Raman and FTIR spectroscopy validated the compositional profiles, identifying silica, carbonates, hydrogen peroxide, and charcoal that explain the observed changes. Overall, charcoal-based dentifrice CNE produced the greatest mineral depletion and heterogeneity, whereas LWE, CBW, and OBW maintained more intermediate or protective effects.

## Graphical abstract

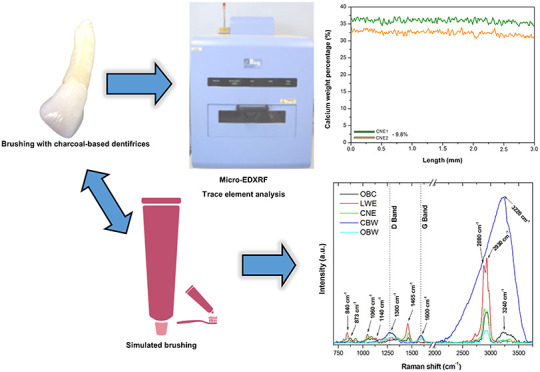

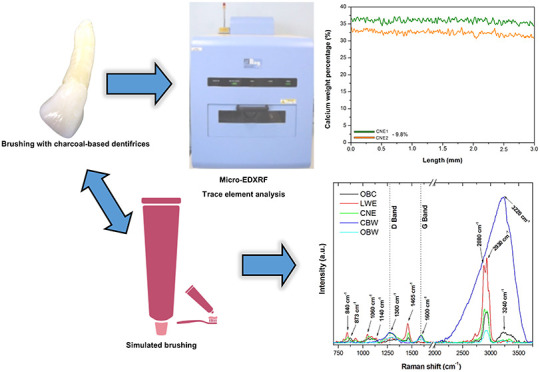

## 1 Introduction

Abrasion is a form of loss of the tooth surface that occurs when external substances come into contact with the tooth surface and slide or rub against it.^1^ The use of abrasive toothpaste, stiff bristles, and a vigorous brushing technique is one of the factors that can cause this loss of tooth structure, leading to clinical presentations such as cervical notches and hypersensitivity seen daily in practice.^1,2^

Pathological loss of tooth structure due to mechanical forces, without the involvement of microorganisms and their products, can occur as a result of wear from excessive brushing habits and the use of highly abrasive dentifrices with a low pH composition.^3^

The abrasive components of dentifrices are the key particles responsible for the physical cleaning (plaque removal) and polishing of the tooth surface.^4^ These components work by mechanically acting on the surface of teeth to remove extrinsic pigments and stains.

Currently, activated charcoal-based dentifrices for tooth whitening are commercialized worldwide. These products are being touted for their ability to whiten teeth, facilitate remineralization, and provide antimicrobial, antifungal, and antiseptic benefits. It is important to acknowledge that their widespread adoption can be attributed to the endorsement of influential figures on social media and the support of manufacturers.^5–7^

Properties, such as high adsorption capacity and porosity, serve as the basis for the incorporation of activated charcoal into dentifrices.^5^ Coal is an insoluble, yet abrasive compound. Activated coal is produced from heated C-rich compounds. During the powder formation process, micropores were created on the surface of C, thereby increasing the adsorption surface. The use of charcoal to clean teeth has been reported for several generations; however, few studies have investigated its benefits and safety.^8^

There are several concerns related to the use of charcoal-based dentifrices that are worth considering. It has been noted that these products do not contain fluoride, which is an essential element for maintaining good oral health.^9^

There is also the possibility that the enamel surface of the teeth could be altered due to abrasion,^10^ and resin composite restorations could experience color change and surface wear.^11,12^ It is also important to note that activated charcoal can absorb the fluoride ion, which can reduce its availability to provide protection for the tooth structure.^13^ Overall, it is essential to weigh the potential risks and benefits of using charcoal-based dentures and to consult with a dental professional for guidance on the best oral care practices.

In light of the growing number of patients who seek to enhance their dental esthetic appearance through the use of tooth-whitening products, it is imperative to assess the effects of charcoal-based dentifrices on dental enamel. A thorough understanding of the mechanisms by which these products operate and their potential impact on our oral health is necessary for making informed decisions regarding our overall dental well-being. Despite the existing research on charcoal dentifrices, no prior study has correlated μ-EDXRF mineral loss with SEM topography under conditions that simulate real-world brushing experiences, leaving a pivotal research gap that this study aims to fill.

Therefore, the current study examined the effects of activated charcoal-based dentifrices on the composition and surface morphology of bovine dental enamel after brushing. The null hypothesis was that there would be no differences in surface and compositional changes of bovine dental enamel after using conventional versus activated charcoal-based dentifrices. Treatment variables were measured using μ-EDXRF and scanning electron microscopy (SEM), both chosen for their ability to provide detailed insights into elemental composition and morphological changes, thus supporting the validity of the methods used in the study. Combined FTIR-Raman analyses were used to evaluate the dentifrice composition and complement the results of the enamel assessments. Additional measurements of surface wettability and enamel roughness complement the pool of analyses performed in total.

## 2 Materials and Methods

### 2.1 Sample Preparation

This study was approved by the Ethics Committee of Universidade do Vale do Paraiba (A7/CEUA/2019). Twenty-five extracted permanent bovine incisors were selected after disinfection in aqueous 0.1% thymol solution (“Nostra Fórmula” Compounding Pharmacy, São José dos Campos, SP, Brazil) for 7 days.^14^

Enamel samples were prepared by sectioning and polishing the bovine samples as described in previous work.^14,15^ Fifty samples were obtained (approximately 7 mm long and 7 mm wide). Finally, the samples were polished with a diamond paste (medium-grain diamond AC I and fine-grain diamond AC II; FGM, Joinvile, SC, Brazil) for 10 s using a polishing machine at 150 rpm (Metaserv 2000; Buehler, Lake Bluff, IL, USA).

### 2.2 Sample Selection via Inorganic Composition Analysis of the Enamel Before Simulated Brushing

The baseline inorganic contents of the prepared enamel samples before treatment were evaluated using μ-EDXRF (model-EDX 1300; Shimadzu, Kyoto, Japan) to standardize the selected samples. Line mappings covered a central region of the selected samples of 300 × 1 points (20 μm steps, 10 s/point, and 15 kV).

Statistical analyses were performed using the Tukey–Kramer multiple comparison test in GraphPad Prism software (GraphPad Software, San Diego, CA, USA). A comprehensive analysis was carried out on the enamel samples in order to gain a better understanding of their characteristics. To ensure the highest level of accuracy, the samples were divided into five groups at random, with each group containing 10 specimens based on their individual treatments. A summary of the treatments and their corresponding groups is given in Table 1.

**Table 1:** Dentifrices Detailed Composition^a^

dentifrice	composition
OBC	Hydrated Silica, Sorbitol, Water, Disodium Pyrophosphate, Flavor, Sodium Lauryl Sulfate, Sodium Hydroxide, Alcohol, Xanthan Gum, Sodium Saccharin, Polyethylene, Glycerin, Carbomer, Poloxamer 407, Titanium Dioxide, Mica, Limonene, Cinnamal, Blue Lake 1, lake 10 yellow, polysorbate 80, iron oxides, sodium benzoate, cetylpyridinium chloride, yellow 5, benzoic acid. Sodium Fluoride (1100 ppm Fluoride).
LWE	Hydrogen Peroxide 2%, Propylene Glycol, Calcium Pyrophosphate, RVP, Hydrogen Peroxide, PEG/PPG-116/Copolymer 66, PEG-12, Glycerin, Aroma, Sodium Lauryl Sulfate, Silica, PVP, Tetrasodium Pyrophosphate, Sodium Saccharin, Sodium Monofluorophosphate, Disodium Pyrophosphate, Sucralose, BHT, Eugenol. 0.76% Sodium Monofluorophosphate (1000 ppm Fluoride).
CNE	Hydrated Silica, Sorbitol, Aqua, Sodium Lauryl Sulfate, Flavor, PEG-12, Tetrasodium Pyrophosphate, Cocamidopropyl Betaine, Cellulose Gum, CI 77891, Mica, CI 77266 (Charcoal Powder), Benzyl Alcohol, Eugenol. Sodium Fluoride (1000 ppm Fluoride).
CBW	Hydrated Silica, Hydroxyapatite, Sorbitol, Glycerin, Carbon Black, Aqua, Aroma, Bentonite, Sodium Monofluorophosphate, Decyl Glucoside, Cocamidopropyl Betaine, Tocopherol, Xanthan Gum, Mica, Potassium, Acesulfame, Maltodextrin, Titanium Dioxide, Sodium Benzoate, Cellulose Microcrystalline, Potassium Sorbate, Potassium Chloride, Lemon Peel Oil, Mentyl Lactate, Methyl Diisopropyl Propionamide, Ethylmentane, Carboxamide, Sodium Hydroxide, Citric Acid, Lacto peroxidase, Glucose Oxidase, Amyloglucosidase, Potassium Thiocyanate, Sucrose, Zea may starch, stearic acid, cetearyl alcohol, tin oxide, hydrogenated lecithin, limonene, CI 75815, CI 77289. Sodium Fluoride (950 ppm Fluoride).
OBW	Hydrated Silica, Sorbitol, Aqua, Disodium Pyrophosphate, Sodium Lauryl Sulfate, Cellulose Gum, Flavor/Flavor, Sodium Hydroxide, Sodium Saccharin, Carbomer, Charcoal Powder, Mica, Limonene, Sucralose, Titanium Dioxide, Polysorbate 80. Sodium Fluoride (1100 ppm Fluoride).

a Legend: OBC, Oral-B complete; LWE, Colgate Luminous White Expert; CNE, Colgate Natural Extracts Purifying; CBW, Curaprox Black Is White; and OBW, Oral-B 3D White Mineral Clean (Bamboo Charcoal).

Samples brushed with conventional toothpaste without bleaching agents (Oral-B Complete [OBC]) comprised the negative control (Group OBC). Samples brushed with traditional whitening toothpaste (Colgate Luminous White Expert [LWE]) were part of the positive control group (Group LWE). The remaining samples were brushed with charcoal-based toothpaste (Colgate Natural Extracts Purifying [CNE], Curaprox Black Is White [CBW], or Oral-B 3D White Mineral Clean [OBW]).

### 2.3 Determination of Baseline Mean Surface Roughness

A roughness tester (TR200; Qualitest International Inc., Ft. Lauderdale, FL, USA) was used to evaluate the *R*_a_ values of the samples. A baseline measurement (first reading) of roughness was performed for all samples (150 roughness profiles) for the statistical comparison of each treatment group. Each sample was measured at three regions separated by a distance of 15 μm.^14^ The average *R*_a_ was obtained by calculating the average of the three profiles per sample. *R*_a_ was used because it reproduced the arithmetic mean of all of the absolute distances of the roughness profile (*R*) from the central line within the measurement range (*L*_m_) (measurement limit = extension considered in the reading). The *L*_m_ considered was 1.25 mm, and the cutoff was 0.25 mm.^14^

### 2.4 Abrasion

To perform the simulated brushing, each dentifrice slurry was prepared by diluting the dentifrice in artificial saliva (“Nostrafórmula” Compounding Pharmacy, São José dos Campos, SP, Brazil) by a weight ratio of 1:3, reproducing the dilution in human saliva. Identical toothbrushes (Medfio, Pinhais, and PR, Brazil) were used, and each group had an associated toothbrush.

Abrasion was performed using an automatic brushing machine (MSE; Elquip, São Carlos, SP, Brazil). The toothbrush head was affixed to the brushing machine holder in a parallel orientation to ensure proper alignment with the enamel surface. A simulated brushing motion was then executed with 2500 reciprocal movements, requiring approximately 9 min and 50 s to complete, in accordance with the preprogrammed settings of the brushing machine.

A slightly shorter brushing time than that recommended for toothbrush replacement (every three months) was evaluated in the present study according to a previously published protocol.^16^ Two months of daily brushing were equivalent to 2500 complete movements.^16^ Thus, 2500 cycles at a speed of 4.5 cycles/s—considering a brushing time of 1 min/day and 5 s/tooth surface—would equate to 9 min of constant brushing of each surface or approximately 60 days of brushing; this is reportedly suitable for manual tooth brushing.^16^ Brushing cycles were performed with a load of 200 g force (gf).^17^

To ensure that the samples were fully immersed in the suspensions after brushing, each sample was individually immersed in an acrylic flask containing 3 mL of diluted toothpaste from its respective group for 1 min 45 s according to a previously established protocol.^18^

### 2.5 Chemical and Mechanical Analyses of the Enamel After Simulated Brushing

After brushing, line mappings were performed with μ-EDXRF (second measurement, 50 mappings) using parameters equal to those of the first measurements. The degree of mineral loss or change in the enamel structure after abrasion was estimated by the difference in the mineral-treated content (MTC) and mineral baseline content (MBC).^15,19^ Mineral variation (MV %) was determined using the following formula

$$\mathrm{MV}\%=\frac{\left[(\mathrm{MTC}-\mathrm{MBC})\right]}{\mathrm{MBC}}\times 100$$

Subsequently, new *R*_a_ profiles of the treated enamel were collected (second measurement, 150 profiles) using the same parameters as those used for the baseline measurements.

### 2.6 Scanning Electron Microscopy (SEM) Morphological Analyses

Two representative samples from each group were subsequently selected and dehydrated using a graded series of ethanol (50, 70, 90, and 100%) for 10 min per step and dried at 37 °C for 24 h.^14^ Subsequently, the samples were vacuum-metalized (2 × 10^–1^ mbar) with a thin layer of Au (10 nm). SEM analysis was performed using an SEM instrument (EVO-MA10; Carl Zeiss VR STM, Oberkochen, BW, Germany), and the samples were examined at an acceleration voltage of 20 kV.

The particulate fraction of each toothpaste was analyzed after mixing 15 g of toothpaste with 45 g of deionized water and centrifuging the mixture for 10 min in a centrifuge at 10,000 rpm. The solid material obtained was then dried at 40 °C in an oven for 4 days. Half of the solid particles were separated for spectroscopy analyses, and the other half were fixed to self-adhesive film on aluminum stubs and vacuum-metalized with Au. The micrographs were taken at 20 kV and a magnification of ×300.

**Complementary pool of analyses after brushing**

### 2.7 Surface Wettability–Contact Angle Characterization

The contact angle measurements were used to assess the wettability of the brushed enamel (first analysis, *n* = 40) using a Drop Shape Analyzer system (DSA 100, Top View Analyzer TVA 100, Krüss). Each sample was affixed to a mobile platform, and a 3 μL water drop was dispensed onto the surface of the sample. The system recorded the kinetics of the drop shape change with three measurements taken for each sample.

### 2.8 μ-EDXRF Surface Area Measurements

As a complementary analysis, μ-EDXRF area mappings were conducted (*n* = 40) to examine a defined surface area following brushing and to determine the elemental distribution of the inorganic components across the enamel surfaces. The following parameters were applied: 20 × 20 points, a step size of 20 μm, a voltage of 15 kV, and an incident beam diameter of 50 μm.

### 2.9 FT-Raman and FTIR Spectroscopy of Dentifrices

To perform Raman spectroscopy characterization, the dentifrice sample was taken directly from the toothpaste tube and placed in an aluminum holder without any prior preparation. The inorganic and organic content of the dentifrice samples (*n* = 5) was analyzed using FT-Raman spectroscopy (RFS 100/S – Bruker Inc., Karlsruhe, Germany). The samples were excited with an air-cooled Nd:YAG laser (λ = 1064.1 nm). The incident laser power on the samples was 150 mW. The spectra obtained ranged from 100 to 4000 cm^–1^. The spectral resolution was set to 4 cm^–1^, and for each sample, one spectrum was collected with 200 scans.

Fourier-transform infrared spectroscopy spectra of all dentifrice samples were collected in the MIR region (550–4000 cm^–1^) with a PerkinElmer spectrometer (PerkinElmer Inc., USA). A sample of each dentifrice was dehydrated for 16 h to perform the FTIR analysis. For each spectrum, the resolution was set at 4 cm^–1^, and the number of scans was 16.

### 2.10 Data Processing and Statistical Analyses

For the qualitative spectral analysis, the FT-Raman and FTIR spectra of the dentifrice powder were baseline-corrected using Microcal Origin 6.0 software (Microcal Software, Inc., Northampton, MA, USA).

Data obtained from μ-EDXRF, roughness, and contact angle analyses were statistically analyzed using the GraphPad Prism software (version 5.01 for Windows, GraphPad Software). Statistical analyses were performed using Dunnett’s multiple comparison test (negative/positive control and treatment comparison) or Bonferroni’s multiple comparison test (treatment comparison).

## 3 Results

### 3.1 Mapping by μ-EDXRF

In-line mappings showed changes in composition after brushing cycles (Figures 1 and 2). Calcium (Ca) content was much lower in the OBC and CNE groups than in the others (Figures 1 and 3). LWE and CBW groups had moderate Ca changes, while OBW had the smallest Ca loss (Figure 1). Ca content decreased in this order: CNE > OBC > LWE > CBW > OBW, indicating a decreasing Ca loss. Phosphorus (P) showed a similar pattern (Figure 3).

**Figure 1:**
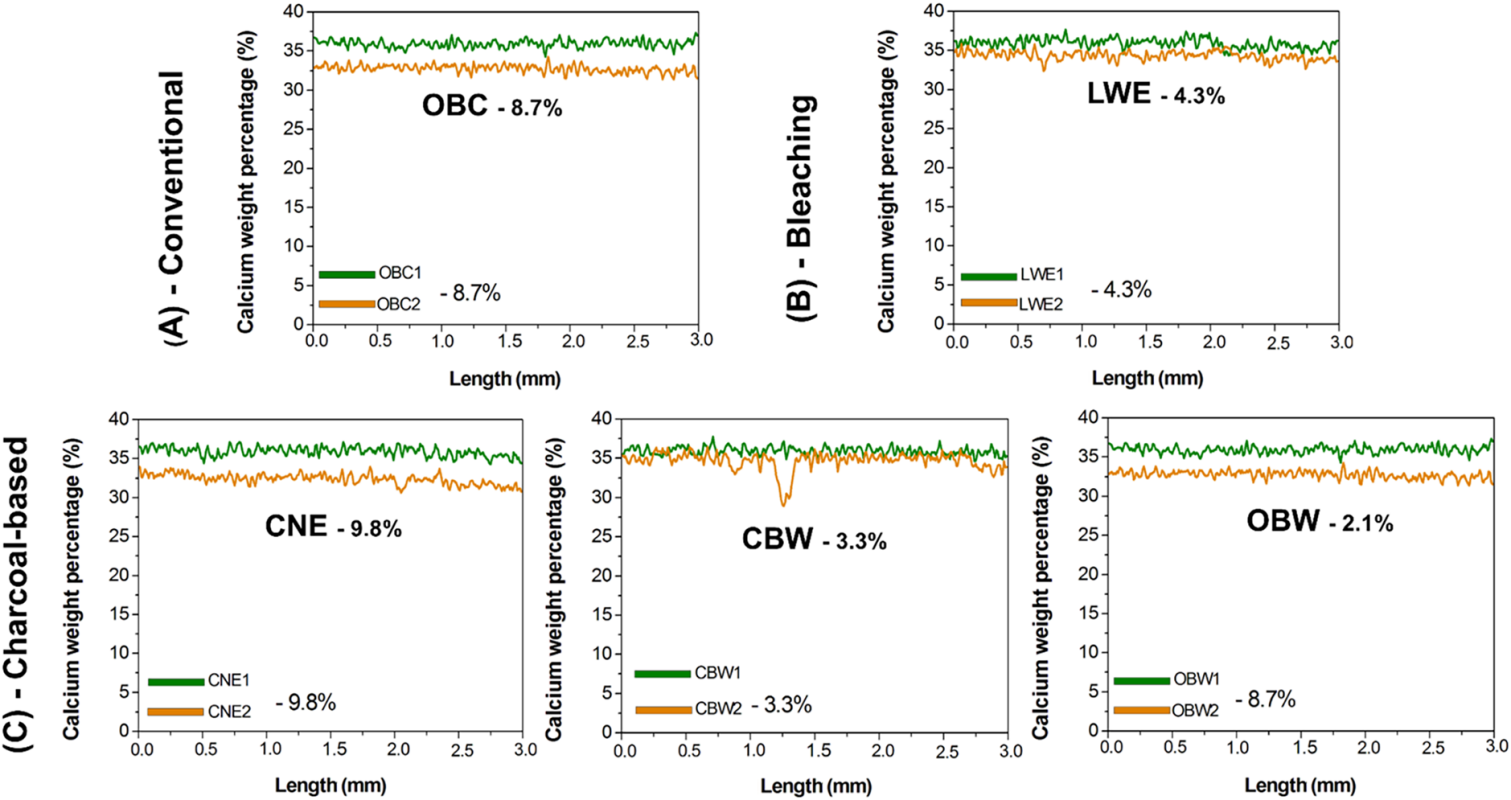
Average curves obtained by μ-EDXRF line-scan in length (mm) for calcium content (%) considering the conventional and whitening dentifrices (A, B) or charcoal-based dentifrices (C). The difference between before and after treatment within the inorganic content was calculated (%). Legend: OBC, Oral-B complete; LWE, Colgate Luminous White Expert; CNE, Colgate Natural Extracts Purifying; CBW, Curaprox Black Is White; and OBW, Oral-B 3D White Mineral Clean.

**Figure 2:**
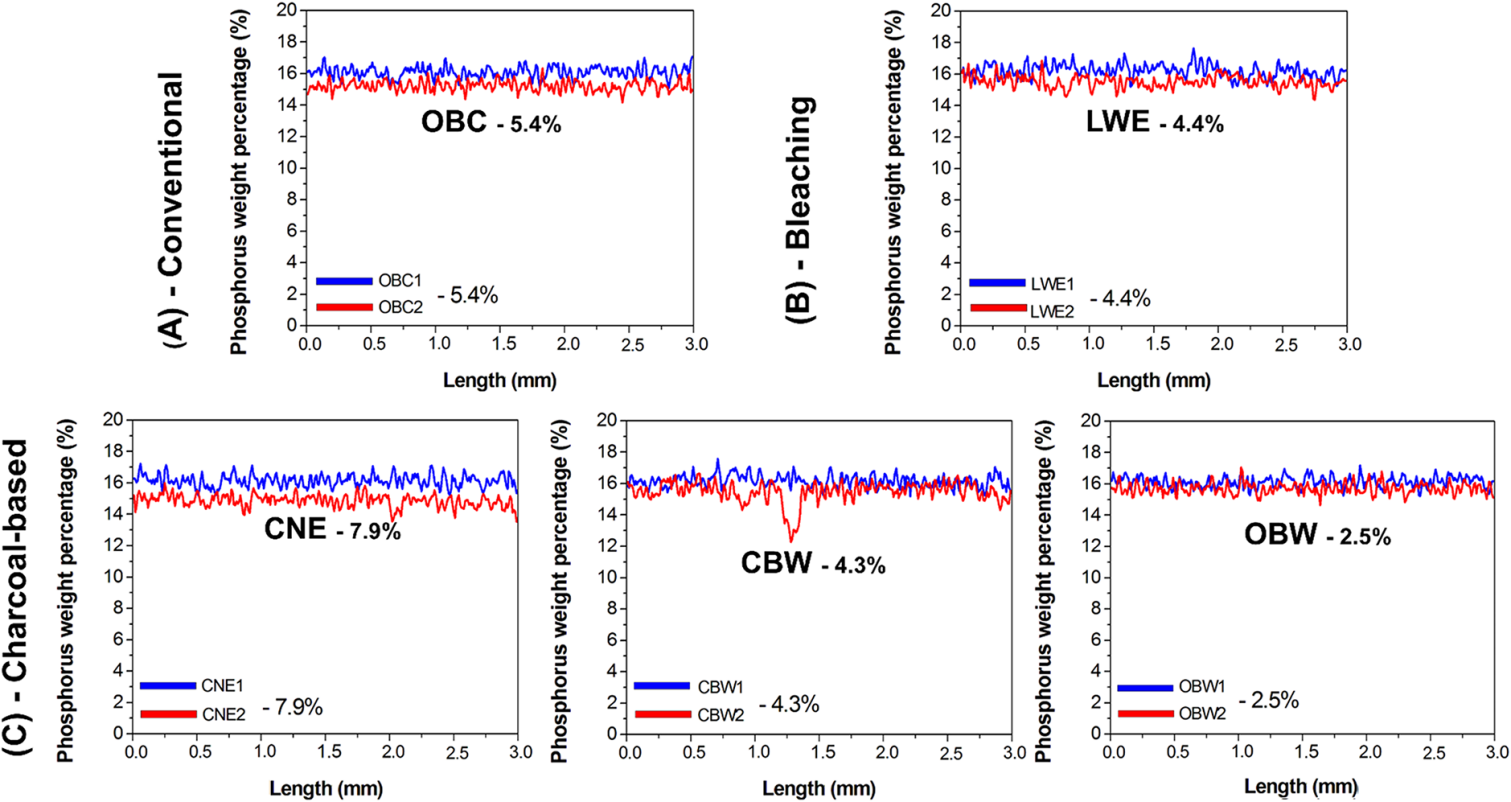
Average curves obtained by μ-EDXRF line-scan in length (mm) for phosphorus content (%) considering the conventional and whitening dentifrices (A, B) or charcoal-based dentifrices (C). The difference between before and after treatment within the inorganic content was calculated (%). Legend: OBC, Oral-B complete; LWE, Colgate Luminous White Expert; CNE, Colgate Natural Extracts Purifying; CBW, Curaprox Black Is White; and OBW, Oral-B 3D White Mineral Clean.

**Figure 3:**
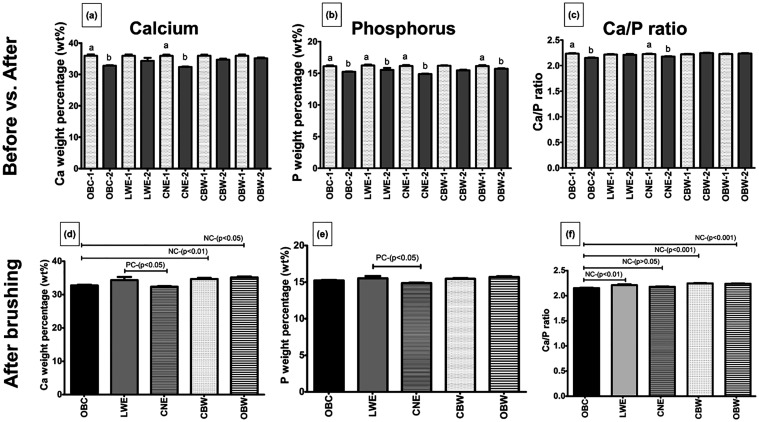
Mean and standard deviation (*n* = 10) of calcium (Ca) and phosphorus (P) weight percentage (%) and molar ratio Ca/P of dentin obtained by μ-EDXRF mapping. Statistical comparisons were performed before and after treatments (a–c) and after treatments (d–f). Bonferroni multiple comparison test: before and after treatments. Different letters represent statistical differences between the first (1-before erosion: light gray bar) and the second measurement (2-after erosion: dark gray bar) (*p* < 0.001) (a–c). Dunnett’s multiple comparison test: between negative control (NC) and treatments, and between positive control (PC) and treatments (d–f).

A statistically significant decrease in Ca content was found in both the OBC and CNE groups (*p* < 0.001) (Figure 3a). Additionally, a significant drop in P content was observed in the OBC and CNE groups (*p* < 0.001), as well as in the LWE and CBW groups (*p* < 0.01), but not in the OBW group (*p* > 0.05) (Figure 3b). The Ca/P ratio showed a significant reduction in the OBC (*p* < 0.001) and CNE (*p* < 0.05) groups, with the most substantial reduction in the OBC group (Figure 3c).

After treatment, the OBW group showed a higher calcium content compared to both the OBC (*p* < 0.01) and CBW (*p* < 0.05) groups (Figure 3d). Conversely, the CNE group had significantly lower levels of Ca and P contents (*p* < 0.05) compared to the LWE group (Figure 3e). Regarding the Ca/P ratio, the OBC group had a lower ratio than the other groups (CBW, OBW, *p* < 0.001; LWE, *p* < 0.01). However, there was no statistically significant difference between the OBC and CNE groups (*p* > 0.05) (Figure 3f).

Analysis of MV% using the baseline data for each group as a reference revealed a significant difference in Ca content between the OBC and OBW groups (*p* < 0.05) (Figure 4). Based on the statistical comparison between the groups that used activated charcoal-containing dentifrice, it was found that the OBW group had a lower MV% for Ca and P compared to the CNE group (*p* < 0.05) (Figure 4)

**Figure 4:**
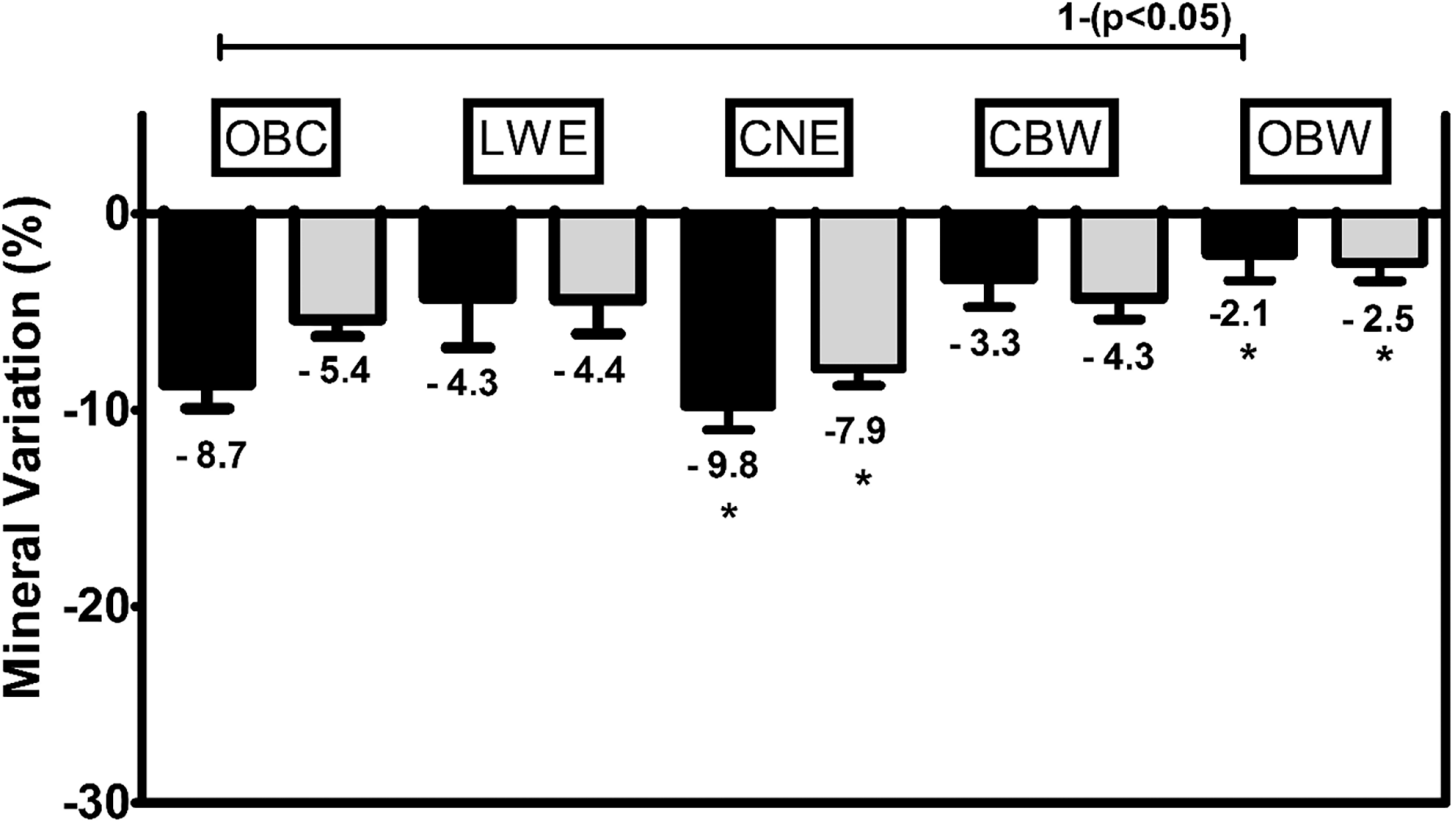
Mineral variation (%) of the enamel (calcium–black bars and phosphorus–gray bars) assessed by μ-EDXRF after treatments (mineral-treated content, MTC) compared to the baseline content (MBC) (as represented by the control group). Statistical comparisons for Ca and P elements (Dunnett’s multiple comparison test); capped line at the top shows statistical significance between negative control and charcoal-based dentifrice; asterisk * denotes statistical significance between treatments (*p* < 0.05).

### 3.2 Surface Profilometry Analyses of Brushed Enamel

The initial mean Ra did not demonstrate a statistically significant difference between the groups (*p* > 0.05) (Table 2). The initial *R*_a_ values ranged from 0.029 (0.006) to 0.034 (0.007). The final *R*_a_ values varied between 0.033 (0.007) and 0.037 (0.006), with no statistically significant differences between the groups. After brushing, the groups exhibited decreasing *R*_a_ values in the following order: OBC≅OBW > CBW > CNE > LWE. The variation in *R*_a_ between the initial and final readings decreased in the following order: OBC > CNE≅OBW > LWE > CBW.

**Table 2:** Means and Standard Deviation of the Mean Roughness Values (*R*_a_) of the Experimental Groups Before and After the Treatments^a^

group	*R*_a_ (μm)–before	*R*_a_ (μm)–after	variation (%)
OBC	0.032 (0.008)	0.037 (0.006)	16
LWE	0.030 (0.011)	0.033 (0.007)	8
CNE	0.029 (0.006)	0.033 (0.008)	11
CBW	0.034 (0.005)	0.034 (0.007)	1
OBW	0.034 (0.007)	0.037 (0.007)	11

a Legend: OBC - Oral-B complete; LWE - Colgate Luminous White Expert; CBW - Curaprox Black Is White; CNE - Colgate Natural Extracts Purificante; OBW - Oral-B 3D White Mineral Clean.

### 3.3 Morphological Analyses of Brushed Enamel

Representative surface micrographs from each group illustrate differences in enamel surface morphology after brushing at two magnifications (Figure 5a–e: 2000×; 5f–j: 5000×). All groups show a loss of surface structure after brushing. At lower magnification, lines from polishing and brushing appear in all groups except the CNE group (Figure 5c). Residual particles remain on the CNE group surfaces (Figure 5c). The CBW (Figure 5d–i) and OBW (Figure 5e–j) samples show less surface degradation.

**Figure 5:**
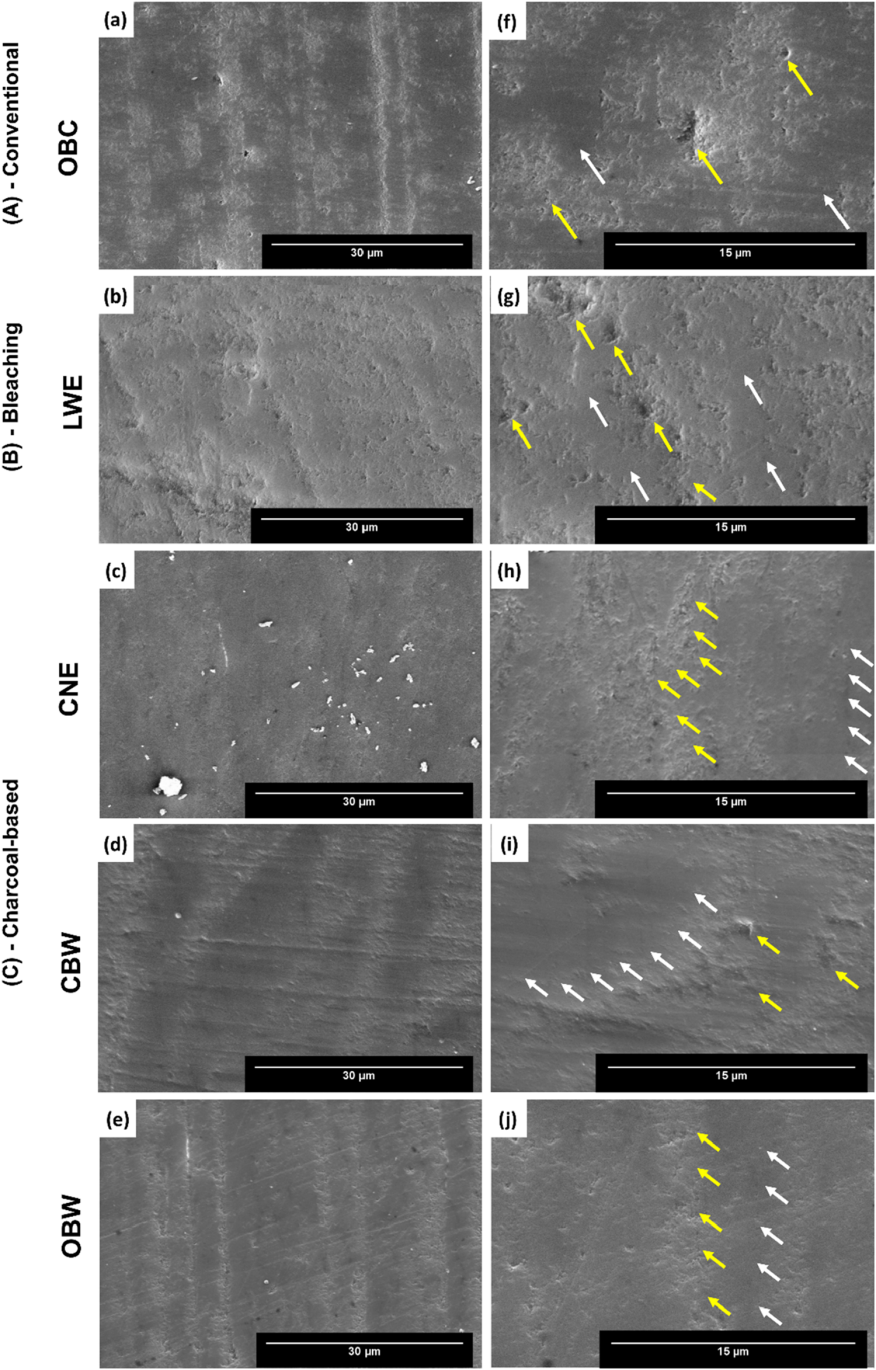
Enamel micrographs after brushing with (A) conventional, (B) whitening, and (C) charcoal toothpaste show differences in enamel surface morphology. Representative images highlight changes after brushing (a–e: 2000×; f–j: 5000×). Structural loss (yellow arrows) and remaining structure (white arrows) are clearer at higher magnification (f–j: 5000×). The OBC (f), LWE (g), and CNE (h) groups showed similar surface modification, while CBW (i) and OBW (j) had less modification (5000×). Magnification bars: 30 μm (2000×), and 15 μm (5000×).

The surface details are better visualized at the highest magnification, where a loss of structure interspersed with the remaining structure can be observed (Figure 5f–j). The samples from the OBC, LWE, and CNE groups exhibited similar patterns of surface modification. The samples from the CBW (Figure 5i) and OBW (Figure 5j) groups had less enamel alteration and a smoother surface texture than those of the other groups.

### 3.4 Morphological Analyses of Dentifrices Particles

The SEM images (Figure 6a–e) revealed isolated or agglomerated amorphous particles of various sizes across all dentifrice formulations, with notable differences among groups. The OBC group exhibited small- to moderate-sized agglomerated particles with undefined shapes and moderately rough surfaces (Figure 6a). The LWE group primarily showed spherical or subspherical silica particles with smooth surfaces (Figure 6b). In the CNE group, micrographs revealed numerous large, isolated angular charcoal particles characterized by sharp edges and heterogeneous sizes (Figure 6c, line circle). The CBW group presented a combination of porous charcoal and hydroxyapatite plate or needle-like crystals (Figure 6d). In the OBW group, micrographs identified agglomerates of irregular charcoal and silica mixed particles with abrasive surface textures (Figure 6e).

**Figure 6:**
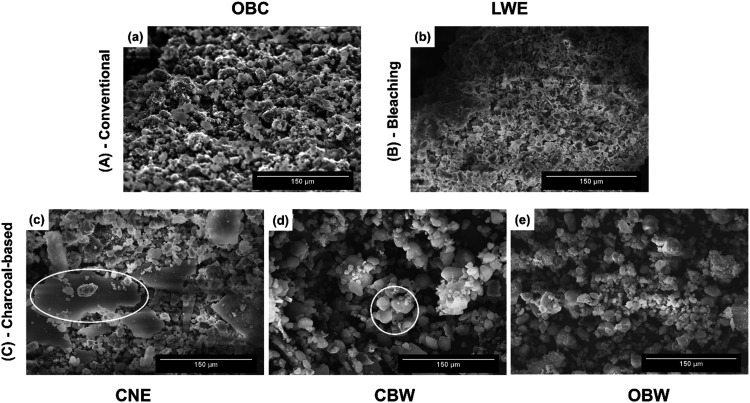
Micrographs of dentifrice inorganic particles OBC (a), LWE (b), CNE (c), CBW (d), and OBW (e), with large and small, circled in CNE and CBW, respectively (300x). Magnification bars: 150 μm.

### 3.5 Surface Wettability–Contact Angle Characterization

The contact angle (CA) values ranged from 90.59° (13.16) in the OBC to 102.6° (13.32) in the CBW. Statistical comparisons of the final CA measurements after brushing revealed no significant differences between the positive and negative controls and the other groups (Table 5).

### 3.6 μ-EDXRF Area Mappings

Figure 7 shows μ-EDXRF area maps of enamel after brushing, with the color scale indicating relative elemental concentration (dark blue = lowest; white = highest). The calcium maps (Ca Kα; max ≈ 30.84 wt %) and phosphorus maps (P Kα; max ≈ 17.56 wt %) reveal distinct spatial patterns across treatments. In the OBC (negative control) maps, the signal is relatively uniform: the central region and upper right quadrant display predominantly intermediate-to-high intensities (green to yellow), while only narrow peripheral bands approach lower intensities (blue), indicating a broadly homogeneous inorganic distribution. The LWE (positive control) maps show a similar homogeneous pattern but with slightly more extensive high-intensity areas in the lower central region, consistent with the preserved surface mineral content.

**Figure 7:**
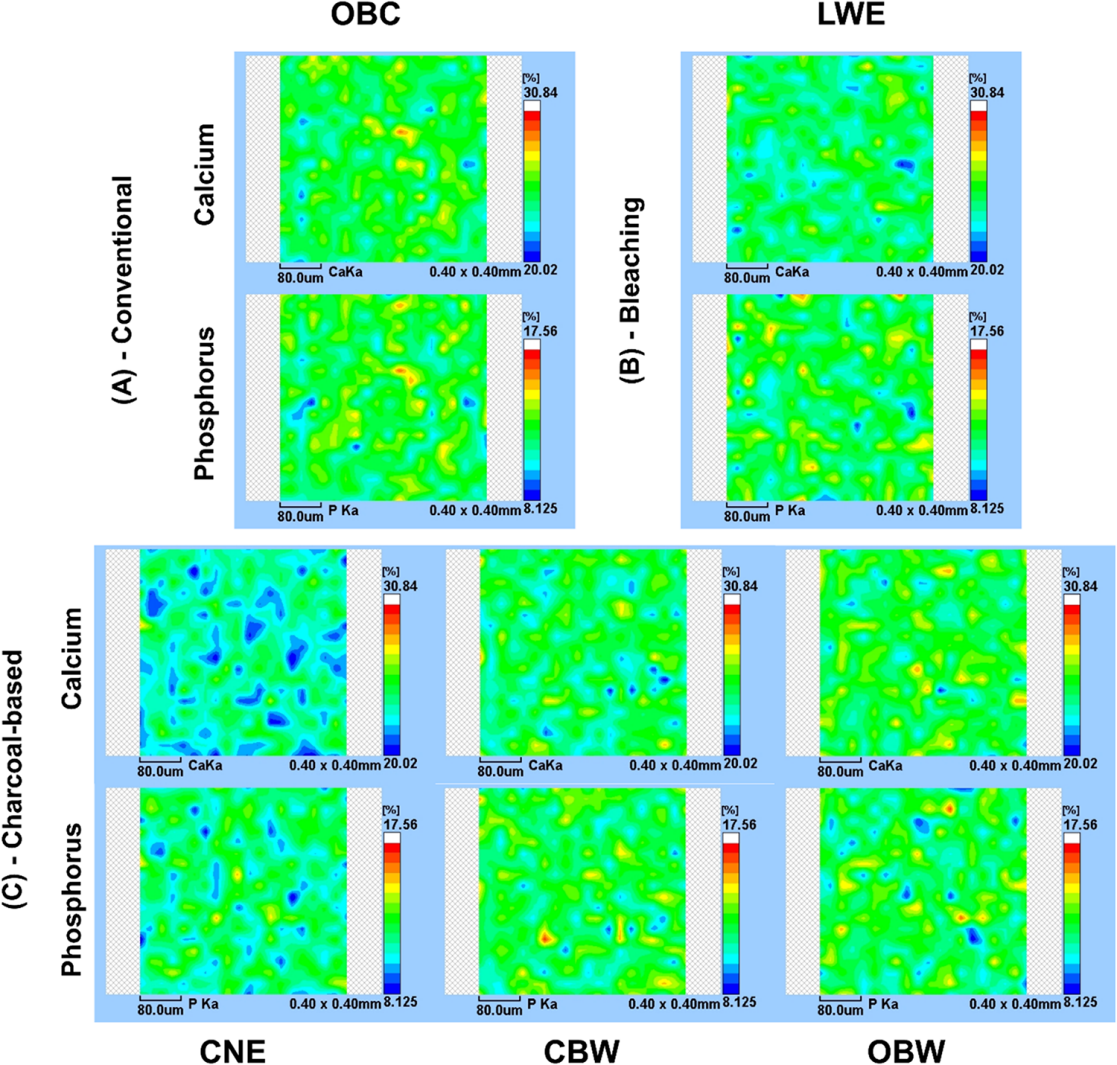
Representative images of calcium (Ca) and phosphorus (P) distribution on the enamel surface obtained by μ-EDXRF microanalysis after simulated brushing in the groups: (A) conventional toothpaste, (B) whitening toothpaste, and (C) charcoal toothpaste. The gradient in the intensity of the color scale indicates variations in Ca and P content, so that areas with high mineral content are shown in red and orange, while areas with low mineral content are shown in green/blue. OBC, Oral-B complete; LWE, Colgate Luminous White Expert; CNE, Colgate Natural Extracts Purificante; CBW, Curaprox Black Is White; and OBW, Oral-B 3D White Mineral Clean. The reduction of phosphorus in the groups occurred in the following order: CNE > OBC > LWE > CBW > OBW.

By contrast, the CNE (charcoal) maps are markedly heterogeneous (Figure 7c). The upper left quadrant and central-left region contain irregular low-intensity patches (dark blue) interspersed with isolated high-intensity spots (white to red) near the lower right quadrant, producing a mottled appearance that suggests focal depletion and localized retention of Ca and P.

The CBW and OBW maps are intermediate: both show generally uniform intermediate intensities across the central and right-hand regions. However, OBW exhibits scattered higher-intensity spots toward the upper central area and slightly increased low-intensity streaks along the left margin, indicating modest spatial variability greater than that of CBW.

### 3.7 FT-Raman and FTIR Spectroscopy of Dentifrices

The complete FT-Raman spectra (750–3750 cm^–1^, with a break in the *x*-axis near 2000 cm^–1^) differentiate fingerprint features (750–1800 cm^–1^) from high-wavenumber C–H and O–H stretching regions (2800–3700 cm^–1^) (Figure 8).

**Figure 8:**
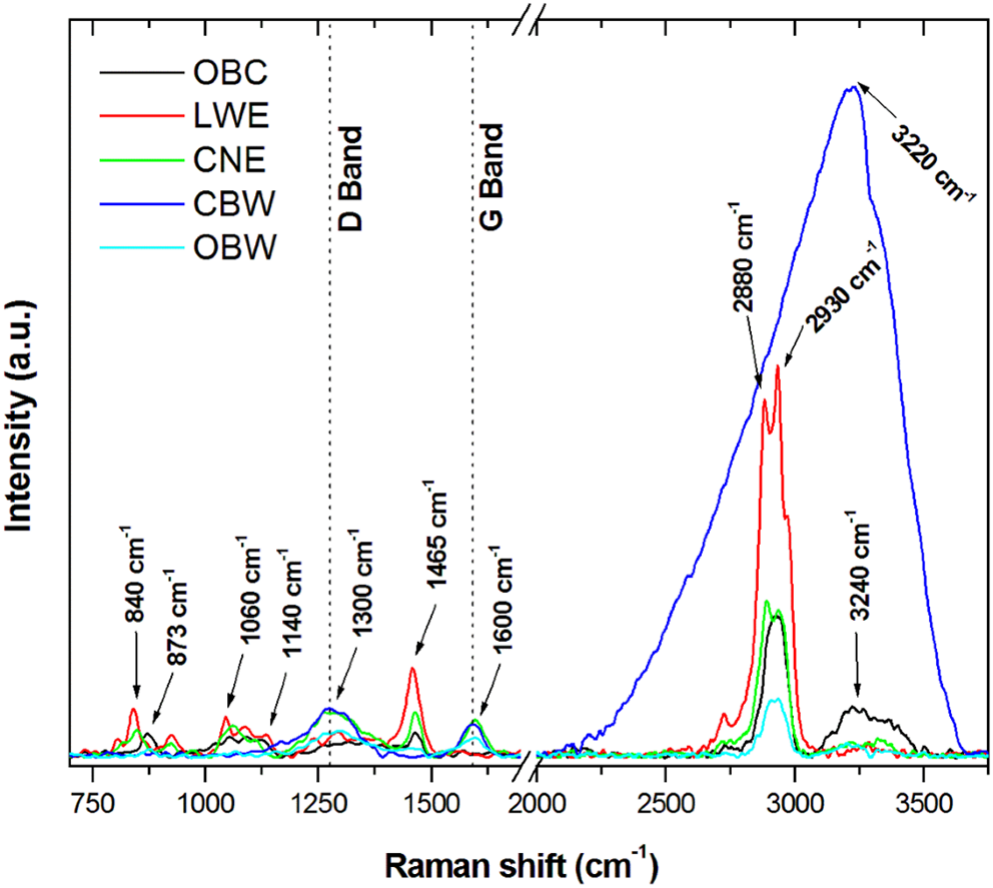
FT-Raman spectrum of the dentifrices with the identification of the main peaks related to the composition. OBC, Oral-B complete; LWE, Colgate Luminous White Expert; CNE, Colgate Natural Extracts Purificante; CBW, Curaprox Black Is White; and OBW, Oral-B 3D White Mineral Clean.

FT-Raman spectra of dentifrices exhibited distinct molecular signatures corresponding to their compositional profiles. In the fingerprint region (750–1800 cm^–1^), LWE and the X-ray microscopy (X-ray) OBC showed peaks at 840 and 873 cm^–1^, respectively, which are attributed to carbonates (CO_3_^2–^).

OBC, LWE, and CNE exhibited from 1060 to 1140 cm^–1^, assigned to SiO_2_. OBC, LWE, and CNE exhibited a peak at ∼1465 cm^–1^ that corresponds to CH_3_ bonds.

Carbonaceous features were prominent in CNE, CBW, and OBW, with broad bands at ∼1350 cm^–1^ (D band) and ∼1580–1600 cm^–1^ (G band), indicative of disordered and graphitic carbon structures from charcoal-based ingredients.

In the high-wavenumber region (2800–3000 cm^–1^), all samples showed aliphatic C–H stretching bands. The peaks at 2880 and 2930 cm^–1^ are related to CH_2_ bonds in glycerin in OBC, LWE, and CBW and polyethylene glycol 12 (PEG-12) in LWE and CNE. The spectrum of the CBW group is dominated by a large peak centered at ∼3240 cm^–1^, which is related to H_2_O bonds.

Figure 9 presents the FTIR spectra of the tested dentifrices. The spectral region between 700 and 1700 cm^–1^ contains the most prominent features, resulting from the overlapping contributions of various ingredients.

**Figure 9:**
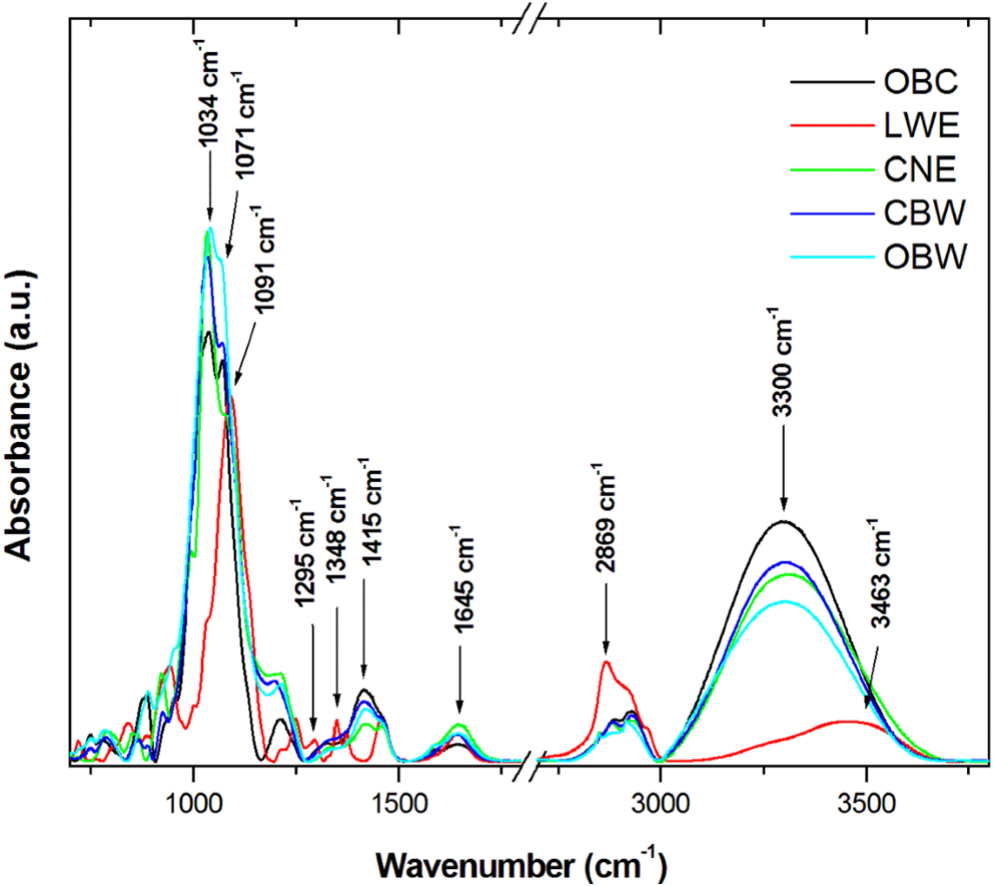
FTIR spectra of the dentifrices with the identification of the main peaks related to the composition. OBC, Oral-B complete; LWE, Colgate Luminous White Expert; CNE, Colgate Natural Extracts Purificante; CBW, Curaprox Black Is White; and OBW, Oral-B 3D White Mineral Clean.

FTIR analysis of the dentifrices revealed characteristic absorptions in the mid-IR region (Figure 9). Strong bands between ∼1030 and 1071 cm^–1^ were observed in all samples except for LWE and are consistent with Si–O stretching from hydrated silica abrasives. The LWE group showed an intense band at 1091 cm^–1^ that is probably related to C–O/C–O–C vibrations from PEG and polyols.

Hydrogen peroxide (H_2_O_2_) shows important FTIR absorption peaks related to the O–H stretching and the O–O stretching exclusive to the LWE dentifrice. The bands at 1295 and 1348 cm^–1^ are representative of O–H deformation vibration, and the band at 2869 cm^–1^ is attributed to the O–H stretching vibration of hydrogen peroxide.

A band at 1415 cm^–1^ suggests the presence of carbonate species, while the 1645 cm^–1^ feature is attributed to H–O–H bending (absorbed water). The broad O–H bands at ∼3300 and 3463 cm^–1^ reflect the organic matrix (glycerine, sorbitol, and PEG) and water content.

## 4 Discussion

In the present study, we used μ-EDXRF chemical analysis to elucidate the influence of inorganic elements in charcoal-based toothpastes on enamel susceptibility to abrasion and loss of minerals. We demonstrated the potential of μ-EDXRF for revealing incipient chemical changes. It is important to emphasize that these initial changes may later evolve into large areas of hypomineralization or demineralization, clinically emerging as white spots.

The chemical profiles obtained via μ-EDXRF analysis revealed significant changes in the inorganic composition of the enamel after brushing, mainly in the OBC and CNE groups (Figures 1–3). The data set demonstrated that brushing with conventional fluoridated dentifrice (OBC) and activated charcoal-based dentifrice (CNE group) resulted in significant changes in enamel composition after a 2-month simulated brushing period.

Our main results revealed that the Ca and P contents and Ca/P ratios were significantly affected after brushing with both conventional (OBC) and activated charcoal-based (CNE) dentifrices (Figures 1–3), thus rejecting our initial null hypothesis. In contrast, the LWE, CBW, and OBW groups showed intermediate patterns of change (Figures 1–3). This suggests that dentifrices, regardless of the activated charcoal presence, can alter enamel composition. Differences in particle morphology between the morphologies of the OBC and CNE dentifrices were observed, with CNE having larger and isolated charcoal particles, while the morphology of the OBC displayed a mixed particle pattern compared to the other groups. This information emphasizes how particle morphology likely influenced our observed results. Additionally, when considering pharmacy or market prices, the OBC and CNE dentifrices had the lowest costs among those tested. Consequently, in the absence of professional recommendations, patients may be more likely to select these cheaper options and potentially face adverse effects after prolonged use.

After brushing, it was found that the P component was significantly reduced in all groups, except for the OBW group (Figure 3b). This suggests that most of the tested dentifrices caused a change in the P composition of the enamel, even at a low level of detection. The loss of P was attributed to the location of P molecules in the hydroxyapatite structure. P ions are more susceptible to chemical disruption of their chemical bonds than Ca because the P molecule is peripherally localized and more unstable.^14,20^

The CNE group exhibited a significant loss of minerals. In contrast to our findings, Vertuan, da Silva, de Oliveira, da Silva, Justo, Zordan, and Magalhães^7^ found that CNE did not enhance erosive tooth wear (measured by a contact profilometer). The differences in the toothpaste composition may explain these results. We used the first version of the CNE toothpaste, which contained 1100 ppm fluoride, whereas Vertuan, da Silva, de Oliveira, da Silva, Justo, Zordan, and Magalhães^7^ used the newest version, which contained 1450 ppm fluoride. This difference in dentifrice composition in the presence of fluoride at elevated concentrations probably contributed to diminishing wear after brushing, thus protecting the enamel surface.

Chemical analysis by μ-EDXRF showed significant changes in the inorganic enamel matrix after the simulated brushing period, mainly in the OBC and CNE groups. The data set (line mapping, area mapping, and MV%) showed, with a statistically significant difference, that brushing with conventional toothpaste, namely, OBC (control group), and with toothpaste containing activated charcoal from the CNE and OBW groups (Figures 4 and 7) results in important changes in enamel composition.

Overall, CNE produced the most pronounced spatial heterogeneity in both Ca and P maps, whereas LWE, CBW, and OBC maintained more continuous, homogeneous color fields. These visual observations align with the quantitative analyses (Figure 7, Tables 3 and 4): CNE showed significant reductions in mean Ca and P, and OBW presented increased spatial variability of P relative to LWE, supporting the interpretation that the charcoal dentifrice produced focal mineral alterations, while some whitening formulations preserved a more uniform inorganic distribution.

**Table 3:** Area Maps Obtained by Micro-EDXRF were Summarized as Weight Percent (% wt) Mean Values, Standard Deviation (SD), and Coefficient of Variation (CV) for Calcium (Ca) and Phosphorus (P)^a^

analyte	group	mean % wt (SD)	CV %
Ca	OBC	24.86 (0.44)	3.86 (0.10)
	LWE	24.76 (1.75)	3.77 (0.12)
	CNE	23.52 (0.45)	3.91 (0.23)
	CBW	24.03 (0.13)	3.86 (0.13)
	OBW	24.01 (0.32)	3.89 (0.12)
P	OBC	12.18 (0.20)	7.31 (0.20)
	LWE	12.15 (0.56)	7.07 (0.30)
	CNE	11.71 (0.21)	7.40 (0.34)
	CBW	11.92 (0.09)	7.29 (0.20)
	OBW	11.83 (0.17)	7.49 (0.36)

a Legend: OBC - Oral-B complete; LWE - Colgate Luminous White Expert; CBW - Curaprox Black Is White; CNE - Colgate Natural Extracts Purificante; and OBW - Oral-B 3D White Mineral Clean.

**Table 4:** Statistical Analyses of Calcium (Ca) and Phosphorus (P) Weight Percent (% wt) Group Comparisons and Coefficient of Variation (CV) Regarding P Element^a^

analyte	comparison	test	significance
Ca	LWE vs CNE	Dunnett	*P* < 0.05
	OBC vs CNE	Dunnett	*P* < 0.05
	CNE vs CBW	Bonferroni	*P* < 0.05
	CNE vs OBW	Bonferroni	*P* < 0.05
P	LWE vs CNE	Dunnett	*P* < 0.05
	OBC vs CNE	Dunnett	*P* < 0.05
	CNE vs CBW	Bonferroni	ns
	CNE vs OBW	Bonferroni	ns
Significant comparisons for CV % of phosphorus, LWE vs OBW			*P* < 0.05

a Legend: OBC - Oral-B complete; LWE - Colgate Luminous White Expert; CBW - Curaprox Black Is White; CNE - Colgate Natural Extracts Purificante; and OBW - Oral-B 3D White Mineral Clean.

In area mapping analysis using micro-EDXRF, the coefficient of variation (CV) parameter is a statistical measure of data precision and reproducibility. It is calculated as the ratio between the standard deviation and the mean of a series of measurements, usually expressed as a percentage.^21^ In the context of micro-EDXRF mapping, which involves measuring the concentration or intensity of elements in thousands of points (pixels) of the sample, the CV is important because it allows for the assessment of heterogeneity or homogeneity, where higher CV values may suggest real heterogeneity of the sample,^21^ as was observed in the case of the OBW group (Figure 7, Tables 3 and 4). This result indicates a greater spatial variability of P after brushing with OBW compared with the positive control LWE, and this could be related to a different pattern of element removal after brushing, despite in-line maps showing that the OBW treatment resulted in the lowest mineral variation (MV%) after brushing (Figure 4).

In addition, the present study measured the surface changes achieved with contact *R*_a_, which showed an average roughness value. An increase in *R*_a_ can result in bacterial adhesion, leading to biofilm formation and accumulation and ultimately causing damage to teeth and the surfaces of restorations.^11^ Thus, it is essential to carefully consider the choice and mode of toothpaste use in maintaining oral health.

The roughness analysis (Table 2) demonstrated that although no statistically significant differences were observed in the mean *R*_a_ after brushing, increases of 16 and 11% in *R*_a_ were observed in the OBC and CNE groups, respectively, which may have occurred owing to the mineral loss observed using μ-EDXRF. However, the OBW group also showed an increase of 11% in *R*_a_, which could have been due to a deposit layer on the enamel surface or a more localized mineral loss (linear pattern), as seen in the SEM analysis of the enamel surfaces (Figure 5j).

Based on the SEM analysis of the enamel surface (Figure 5) in this study, significant surface changes were observed after the simulated brushing, especially in the OBC and CNE groups. These findings support the results from μ-EDXRF (Figures 1–4 and 7) and the particles’ morphology (Figure 6a,c). In the CNE group, the lack of visible lines on the enamel surface (Figures 5c,h) probably indicates increased wear and mineral loss. The presence of pronounced, uneven grooves and wider furrows in the enamel (Figure 5h), along with irregular porous charcoal granules with very rough surfaces seen in the SEM micrographs (Figure 6c), further supports the EDXRF findings.

The LWE group exhibited an intermediate pattern of surface alteration. The CBW group exhibited a minor change in its morphology. The lowest MV% among all groups occurred in the OBW group, which can be attributed to the more linear pattern of surface degradation observed in the SEM micrographs (Figure 5j).

Larger, heterogeneous charcoal particles (CNE) create localized high-stress impacts that form pits and embed fragments; smaller, uniform silica (CBW, OBW) distributes energy, producing widespread but shallow abrasion.

Previous studies have reported varying results regarding the abrasion pattern generated on the enamel surface using dentifrices containing activated charcoal or charcoal in powder form.^22–24^

SEM analyses performed by Vural, Bagdatli, Yilmaz, Çakır, Altundaşar, and Gurgan^23^ revealed enamel surfaces with little wear and marks attributed to the sample preparation process. Franco, Uehara, Meroni, Zuttion, and Cenci^22^ utilized SEM analysis to explore the effects of powdered charcoal brushing on enamel surfaces. Their findings indicated that the enamel surface did not undergo any noticeable changes after the use of powdered charcoal. In contrast, Silva, Maia, Mitraud, Russo, Poskus, and Guimarães^24^ conducted a study on the impact of whitening dentifrices (LWE and OBW) on the enamel surface, revealing some changes, such as the emergence of more visible enamel prisms. These results suggest that different teeth whitening methods may produce distinct effects on the enamel surface. They attributed these changes to the presence of intermediate or moderate abrasive ingredients (hydrated silica and titanium dioxide) in the whitening dentifrices.

In this study, we found that the phosphorus content in enamel and its surface morphology were significantly affected by brushing with LWE. The influence on phosphorus is likely due to the peripheral localization of phosphorus ions within the hydroxyapatite molecule. We also believe that calcium ions may be affected by the continuous use of this product.

SEM results indicate that particle morphology and composition are primary determinants of enamel surface alterations following simulated brushing. The hydrated silica alone product OBC resulted in substantial mineral loss and produced a deep, polishing-like abrasion pattern. Charcoal-containing formulations generated deeper grooves and pits, consistent with the presence of larger, sharper, and more porous carbon particles. The hydroxyapatite-containing formulation CBW exhibited moderate abrasive effects and probably deposited plate-like mineral phases that filled prism gaps, indicating a partial remineralizing effect. Therefore, the hydroxyapatite-containing formulations may offer an advantage by balancing stain removal with enamel preservation.

The analyses of the physical changes observed in enamel after brushing complemented the other data. This can be observed through the contact angle analysis (CA), which reflects the surface features after a brushing process, where the hydrophilic surface is considered wettable, and the hydrophobic surface is repellent.^25^ The contact angle analysis also showed no statistical differences between groups, despite the CBW group showing the highest CA, with the enamel surface tending to be hydrophobic and smoother, probably due to a polishing effect on the enamel surface (Table 5).

**Table 5:** Results of Comparative Analyses of the Enamel Contact Angle (CA) After Brushing with the Average (*n* = 8) and Standard Deviation (SD) in the Experimental Groups^a^

Group	CA (SD)
OBC	90.59 (13.16)
LWE	99.95 (16.52)
CNE	94.08 (12.28)
CBW	102.6 (13.32)
OBW	91.83 (12.58)

a Legend: OBC - Oral-B complete; LWE - Colgate Luminous White Expert; CBW - Curaprox Black Is White; CNE - Colgate Natural Extracts Purificante; and OBW - Oral-B 3D White Mineral Clean.

FT-Raman spectroscopy provided complementary insights into the molecular composition of dentifrices, confirming the presence of specific inorganic and organic components (Figure 8). FT-Raman spectroscopy measurements were performed on the formulations directly and without any type of sample preparation. In the fingerprint region (750–1800 cm^–1^), carbonate (CO_3_^2–^) peaks at 840 cm^–1^ (LWE) and 873 cm^–1^ (OBC) were identified,^26^ consistent with carbonate abrasives that contribute to cleaning efficacy but may also increase abrasivity.

Broad bands between 1060 and 1140 cm^–1^ observed in OBC, LWE, and CNE correspond to SiO_2_,^27^ although overlap with poly­(ethylene glycol) (PEG) or surfactant vibrations cannot be excluded.

Carbonaceous features were prominent in CNE, CBW, and OBW, with broad bands at ∼1350 cm^–1^ (D band) and ∼1580–1600 cm^–1^ region (G band), indicative of disordered and graphitic carbon structures from charcoal particles.^28^ In the high-wavenumber region (2800–3000 cm^–1^), all spectra exhibited aliphatic C–H stretching bands.^29^ Doublets at 2880 and 2930 cm^–1^ in the OBC, LWE, and CBW corresponded to glycerine and to the poly­(ethylene glycol) 12 (PEG-12) component present in the LWE and CNE formulations.

The identification of PEG-based surfactants is noteworthy, as these compounds are not always highlighted in product labeling and have been associated with mucosal irritation and potential toxicity during long-term exposure.^30^ Also, PEG-12 manufacturing involves potentially toxic ethylene oxide, leading to some safety concerns.

Overall, FT-Raman spectroscopy corroborated the compositional data (Table 1), linking spectral features to specific components such as silica, carbonates, charcoal, and PEG derivatives. These findings strengthen the interpretation of enamel changes observed by μ-EDXRF and SEM, demonstrating how dentifrice composition directly influences both chemical and morphological outcomes.

The FTIR spectra (Figure 9) corroborate the compositional differences among the formulations. The dominant Si–O/C-O envelope (1030–1090 cm^–1^) supports the role of silica abrasives and PEG/polyol excipients in all dentifrices.^31^ The carbonate/carboxylate signal at 1415 cm^–1^ and the pronounced O–H stretching in charcoal-containing products indicate higher organic/water retention and possible carbonate-based abrasives,^32^ which may contribute to the observed enamel heterogeneity and mineral loss. In addition, FTIR identified hydrogen peroxide bands (1295, 1348, and 2869 cm^–1^)^33^ in the LWE product, thus complementing the Raman analysis. Water content and hydration were identified in bands like 1645 and 3300 cm^–1^.^34^

The combined FTIR-Raman data set strengthens the link between formulation chemistry and enamel outcomes. Spectral agreement in the Si–O/C–O region confirms that silica abrasives and PEG/polyol excipients are ubiquitous across samples and likely drive the mechanical abrasion patterns observed by SEM and the mineral loss mapped by μ-EDXRF. Carbonate/carboxylate signatures (FTIR 1415 cm^–1^; Raman 840–873 cm^–1^) identify formulations with carbonate-based abrasives that can increase enamel solubility.

The concurrence of FTIR O–H bands and Raman water/O–H features in CBW indicates higher hydration/polyol retention, which may explain its distinct wettability and surface behavior. Carbonaceous D/G bands in the Raman spectrum, together with FTIR evidence of aromatic and CH deformation bands, confirm the presence of charcoal particles in CNE and OBW and rationalize the focal, heterogeneous mineral depletion seen in μ-EDXRF maps. The detection of hydrogen peroxide bands in FTIR for LWE further clarifies its oxidative component and supports its distinct chemical interactions with enamel. Overall, the complementary vibrational information provides a coherent molecular basis for the observed morphological and chemical enamel changes.

Despite the valuable insights provided by this study, limitations must be considered. In a real clinical situation, dental enamel is subjected to dynamic forces and contact points that cause additional wear patterns compared to only tooth brushing. In addition, the present study focused on only five specific toothpaste formulations, thus limiting the findings. The market for whitening and charcoal-based dentifrices is full of other brands with distinct compositions.

The findings of this study are highly significant for dental professionals, as they offer valuable insights into the composition of dentifrices and their effects on enamel. The results obtained from the μ-EDXRF and SEM analyses can be used to recommend the most suitable dentifrices for patients based on their individual needs and requirements.

Based on the limitations of this in vitro study, we conclude that the CNE toothpaste led to significant morphological changes in the enamel surface, attributed to activated charcoal (C) in its composition. The conventional OBC toothpaste also showed notable changes in the enamel due to the composition of its abrasive agents. In contrast, LWE, CBW, and OBW toothpastes showed intermediate levels of modification patterns. The μ-EDXRF technique demonstrated accuracy for assessing mineral loss after brushing and detecting incipient changes not observed using *R*_a_. FT-Raman and Fourier-Transform Infrared (FTIR) spectroscopy brought relevant information regarding material composition and its correlation with enamel abrasion or modification. The selection and use of a dentifrice are crucial factors in managing oral health, and consequently, recommendations should be made by dental professionals. It is important to emphasize that these findings may not directly apply to clinical situations, and further research is necessary to fully understand the potential benefits and risks of charcoal-based toothpastes for oral health.

## 5 Conclusions

The present study showed that dentifrice formulation strongly influences enamel chemistry and morphology. μ-EDXRF revealed significant mineral loss with the OBC and CNE, while SEM confirmed deeper abrasion patterns associated with larger heterogeneous particles. FT-Raman and Fourier-Transform Infrared (FTIR) spectroscopy validated the compositional profiles of dentifrices, identifying silica, carbonates, hydrogen peroxide, and charcoal that explain the observed changes. Overall, charcoal-based dentifrice CNE produced the greatest mineral depletion and heterogeneity, whereas LWE, CBW and OBW maintained more intermediate or protective effects. These findings highlight the importance of professional guidance in dentifrice selection to balance cleaning efficacy with enamel preservation.
